# Modeling of the Particle Abrasion Process and a Discrete Element Method Study of Its Shape Effect

**DOI:** 10.3390/ma17163947

**Published:** 2024-08-08

**Authors:** Zhengbo Hu, Junhui Zhang, Xin Tan, Hao Yang

**Affiliations:** 1 National Engineering Laboratory of Highway Maintenance Technology, Changsha University of Science & Technology, Changsha 410114, China; 2 College of Civil Engineering, Hunan University, Changsha 410082, China

**Keywords:** granular modeling, abrasion process, mechanical property, fabric anisotropy

## Abstract

This study introduces a novel method for particle abrasion derived from fundamental natural phenomena and mechanical principles, allowing precise control over the degree of abrasion and more accurately mimicking natural processes. The method’s validity is confirmed using a specific shape index. Through conventional triaxial tests, the mechanical behavior of granular aggregates with varying degrees of abrasion was analyzed. The findings indicate that increased particle abrasion leads to a decrease in the average coordination number and sliding amount, while the rotation amount increases. This suggests an inverse relationship between the degree of abrasion and the structural stability and interlocking of the particle aggregate. The fabric anisotropy of the system is mainly attributed to the anisotropy of the contact normal force, which decreases as particle abrasion increases. The partial stress ratio of the particle system is influenced by fabric anisotropy and remains independent of particle shape. Additionally, the internal friction angle may be overestimated in conventional triaxial tests.

## 1. Introduction

Rock and soil particles formed in upstream rivers or by blasting have irregular shapes and sharp angles. Most pebbles in downstream rivers, after long-term scouring and abrasion by water flow, become ellipsoidal and have smooth surfaces. Since differences in particle morphology can lead to variations in the mechanical properties of their aggregates, accurate particle construction in numerical simulations is crucial to ensuring the reliability of the results.

Researchers have confirmed that aggregate shape significantly affects the mechanical properties of granular materials, such as shear strength, deformability, and breakage effects, through both indoor experiments [1,2,3,4,5,6] and numerical methods [7,8,9,10]. Since spherical particles do not reflect interlocking and occlusion between particles, many researchers have developed various methods to generate non-spherical granular aggregate models for DEM simulations. Some researchers used polygons and polyhedra to represent granular materials [11,12,13,14], while others have built two-dimensional ellipsoidal particles [15,16] and expanded these to three-dimensional ellipsoidal shapes [17,18]. Compared with spherical particles, these shapes can partially reflect the interlocking effect between particles. Additionally, granular aggregates with various aspect ratios have been simulated using a spherocylindrical model. However, these models do not fully capture the surface characteristics of the particles. Therefore, some scholars have proposed more complex particle models. In some cases, the surface properties of particles can be reflected by two-dimensional superquadratic curves based on implicit function expressions [19], and three-dimensional superquadratic surfaces have been used to simulate particles with random forms by improving the expressions of the superquadratic surface functions in each quadrant [20,21,22]. These methods, however, do not adequately capture particle asymmetry. Additionally, by using Fourier spherical harmonic functions, some researchers [23,24,25] have created complex particles with asymmetric shapes, but the resulting concave surfaces complicate particle contact identification. Wang [26] proposed a random generation technique for 3D convex aggregates to investigate the impact of particle shape on DEM simulations. This approach involves constructing a curve skeleton using Bezier curve fitting and generating a solid surface using the Gregory surface interpolation algorithm. Nonetheless, the practical implementation of this method can be intricate, and achieving local continuity control and refinement can be challenging. With the help of laser or CT scanning equipment, researchers can extract real particle shapes [27,28,29]. However, these irregular particles require a large number of overlapping spheres to simulate surface angles and depressions, making them inefficient for large-scale calculations. In general, algorithms for generating non-spherical aggregates with random shapes remain limited in DEM simulations.

This study introduces a particle generation method for convex polyhedrons based on spheres and develops a particle abrasion function utilizing mesh segmentation. Using this function, particles with varying degrees of abrasion were generated, effectively simulating the shape changes in particles in river channels due to continuous tumbling and collisions under the influence of water flow, thereby replicating the natural process of abrasion. Additionally, triaxial tests were conducted on particles with different abrasion levels to elucidate the impact of particle morphology on mechanical properties from a microscopic perspective.

## 2. Materials and Methods

### 2.1. Polyhedron Particle Generation Algorithm

**Figure 1:**
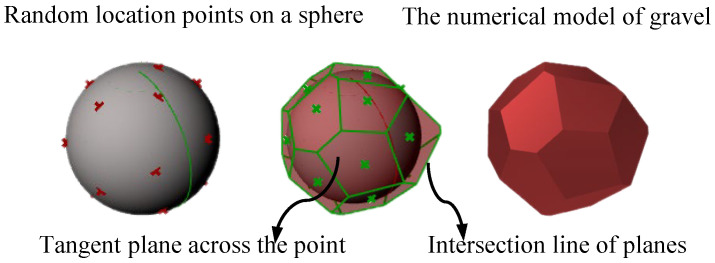
Modeling principle of a polyhedron particle.

A particle generation method with a particular shape index is suggested in this study to simulate the randomness and angularity of the gravel particles. Figure 1 shows the modeling method of convex polyhedron particles with random shape. First, a sphere with radius *R* is created in space at the origin. The sphere’s equation is:

(1) $$F(x,y,z)=x^{2}+y^{2}+z^{2}-R^{2}$$

Assuming that any random point on the surface of the sphere has the coordinates (*x_i_, y_i_, z_i_*), the equation of the plane that is tangent to the surface of the sphere and passes through this random point is:

(2) $$x_{i}x+y_{i}y+z_{i}z=R^{2}$$

As shown in Equation (3), the *N*-hedron formed by the *N* tangent points $\left\{p_{i}\;|\;i=1,\ldots ,N\right\}$ can be expressed as a set of inequality sets if there are *N* random points on the sphere.

(3) $$P=\left\{(x,y,z)|\;x_{i}x+y_{i}y+z_{i}z\leq R^{2},\;i=1,\ldots ,N\right\}$$

**Figure 2:**
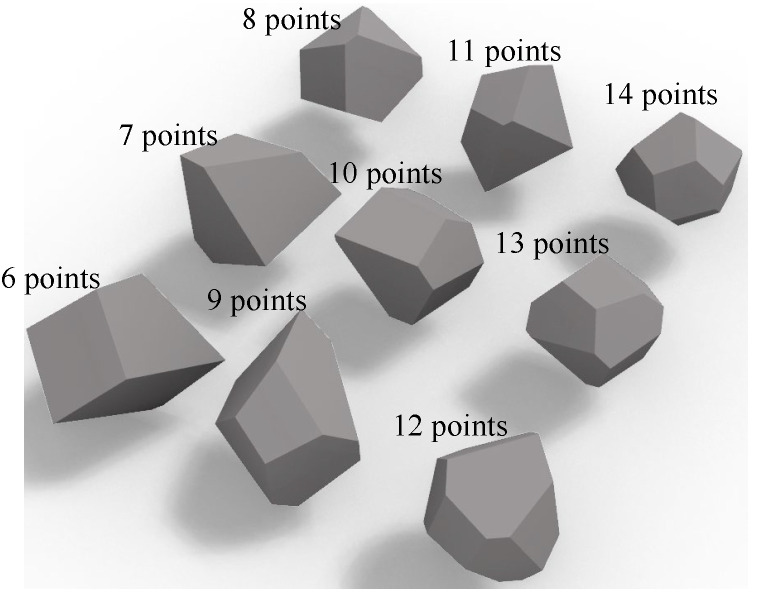
Polyhedron particle template.

Convex polyhedrons take on different shapes as a result of the random positioning of the points on the sphere, better reproducing the variety of particle shapes. In general, the degree of angularity of the particle surface gradually decreases as the number of faces of the convex polyhedron increases. Roundness can be used to reflect the deviation in particles from a standard sphere; the smaller the roundness, the greater the deviation, and the closer it is to 1, the more similar it is to a standard sphere. The number of points on the spherical surface can also be infinite; when the convex hull’s face count is sufficient and the particle’s theoretical roundness is 1, the particle is roughly spherical. Ma [30] indicated that 0.7 is the most likely roundness for an angular particle. As a result, the random convex polyhedron examined in this study has 6~14 faces, as shown in Figure 2.

### 2.2. Particle Abrasion Method

**Figure 3:**
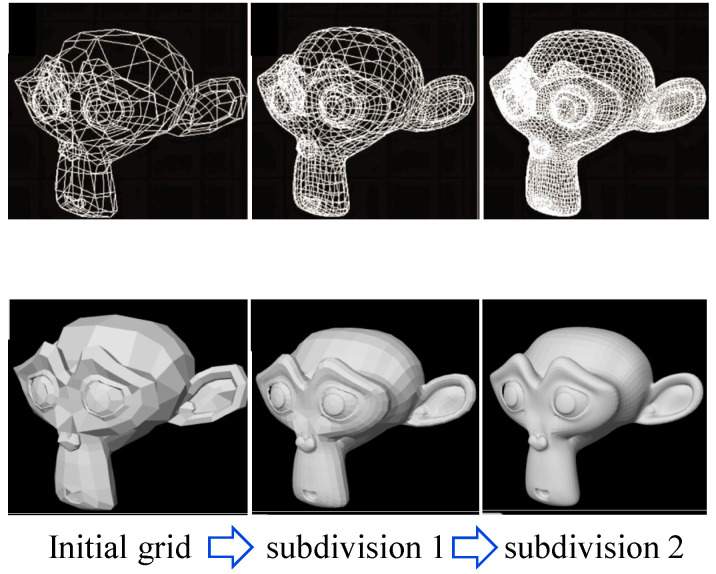
The application of the Catmull–Clark subdivision method in computer modeling.

As shown in Figure 3, the Catmull–Clark subdivision algorithm is widely used in computer graphics to create smooth surfaces from coarse polygonal meshes [31,32]. Its primary function is to iteratively refine a mesh, transforming it into a smoother, more visually appealing surface. This is achieved by calculating new points for faces, edges, and vertices in each iteration and then subdividing the original faces using these points. The algorithm is particularly valued for its ability to handle complex topologies and produce surfaces that are smooth and continuous, making it a standard tool in 3D modeling and animation for generating high-quality, realistic shapes.

The algorithm operates iteratively, refining the mesh at each step to produce a smoother result. The process begins with the initial mesh, where each face, edge, and vertex is considered. In each iteration, the following steps are performed:

1. Calculate the new face point, noted as *f*-point, which is the average of all vertex positions of the defined face, according to Equation (4).

(4) $$f=\frac{1}{n}\sum_{1}v_{i}$$

2. Calculate the new edge point, denoted as *e*-point, defined as the average of the two endpoints of the edge and the two *f*-points of the face sharing the edge, according to Equation (5).

(5) $$e=\frac{v_{1}+v_{2}+f_{1}+f_{2}}{4}$$

**Figure 4:**
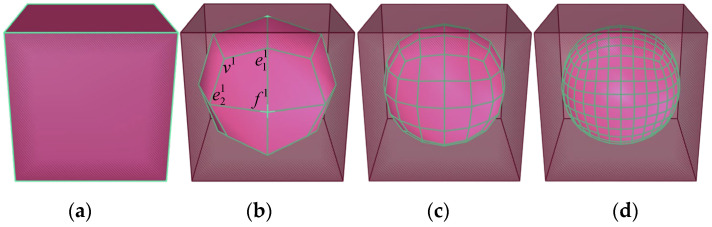
The abrasion process of cube mesh. (**a**) Initial cube mesh; (**b**) after 1 abrasion; (**c**) after 2 abrasions; and (**d**) after 3 abrasions.

3. Calculate the new vertex point, denoted as *v*^1^ point, defined as the vertex generated for each interior vertex of a given mesh; the new vertex is a linear combination of adjacent face points, adjacent edge midpoints, and vertices, calculated according to the equation:

(6) $$v^{1}=\frac{1}{n}Q+\frac{2}{n}R+\frac{n-3}{n}S$$

where *Q* is the average of the new face points sharing the initial vertices. *R* is the midpoint average of all polyhedron edge positions calculated last time that incident on the polyhedron vertex position calculated last time; *S* indicates the old vertex; and *N* is the order of the vertex. Taking the ortho-hexahedral mesh shown in Figure 4 as an example, after generating new vertices, the boundaries are generated in the following order: $v^{1}\to e_{1}^{1}\to f^{1}\to e_{2}^{1}\to v^{1}$. The grid edge is represented by green lines, and the ortho-hexahedral is approximated to a sphere after 3 abrasions. It can be found that the method in this study corrodes the mesh vertices the most, the edges the second, and the surface the least, which can better reflect the abrasive process of particles in nature.

**Figure 5:**
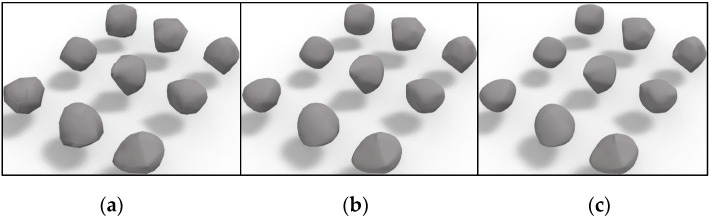
Polyhedron particle template under different abrasion degrees. (**a**) After 1 abrasion; (**b**) after 2 abrasions; and (**c**) after 3 abrasions.

Figure 5 shows the particle models of the nine convex polyhedron particles in Figure 2 formed after 1 to 3 abrasions, respectively. (In the following study, the initial polyhedron particles will be represented by I_P, and the particles after 1 to 3 abrasions will be represented by Ab_1, Ab_2, and Ab_3, respectively.) The randomly convex polyhedron particles go through abrasion, which is akin to the abrasion process of the hexahedral mesh, to reduce the angularity to some extent and make the particles more rounded overall. The rounding of the particles after abrasion is faster for particles with few faces. Furthermore, it should be noted that this abrasion process is infinite. However, a grid that is too dense will reduce the computational efficiency of numerical simulation; therefore, only 1 to 3 abrasions are examined in this study.

### 2.3. Particle Shape Quantification

**Figure 6:**
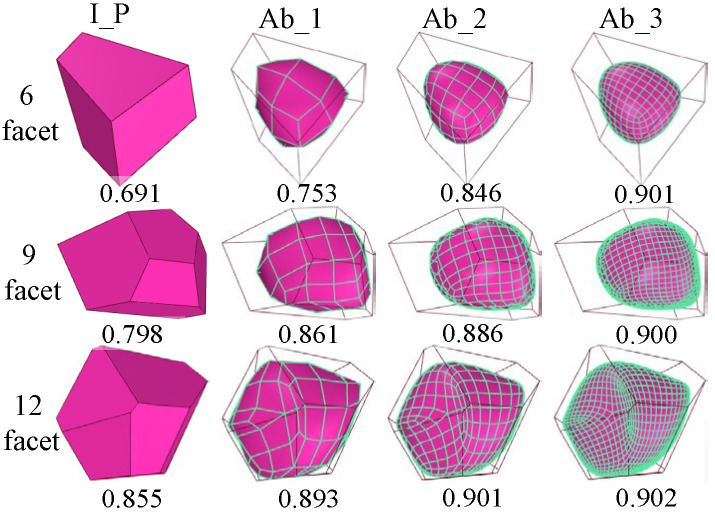
The roundness of initial particles with different numbers of faces after abrasion calculations.

**Figure 7:**
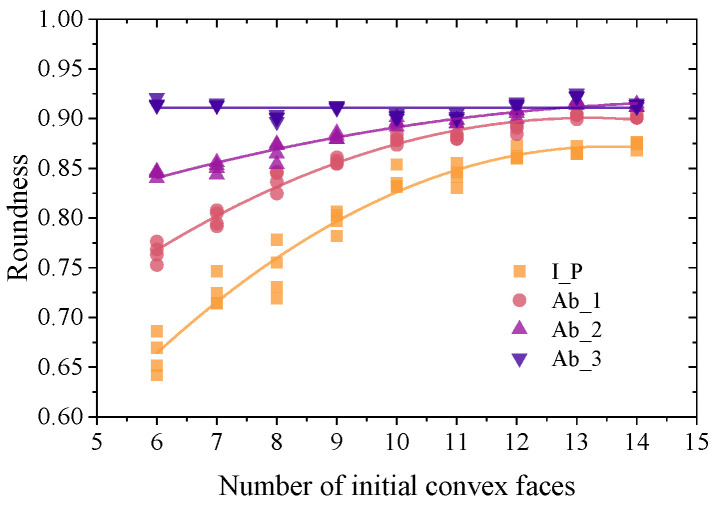
Influence of the surface number and abrasion degree on the roundness of particles.

According to Barrett’s [33] definition, Equation (7) can be used to calculate the roundness evaluation index of particle shape. This evaluation method is straightforward and can more accurately reflect the degree of deviation between particles and standard spheres. *A*_s_ represents the surface area of a sphere equal in volume to the particle; *A_p_* and *V_p_* stand for the particle’s surface area and volume, respectively. Figure 6 illustrates how the number of surfaces after abrasion affects the roundness of the polyhedron particles. After abrasion, the roundness of the initial polyhedron particles with varying numbers of surfaces increases. The roundness statistics of convex polyhedron particles with various numbers of surfaces after four regenerations are shown in Figure 7 in order to make the findings more universal. When there are fewer than ten surfaces, the polyhedron’s roundness increases quickly with the number of surfaces; however, when there are more than eleven surfaces, the roundness of the particles almost no longer increases with the number of surfaces. The dispersion of the distribution of roundness increases as the surface number of polyhedrons decreases. Following abrasion, the dispersion gradually decreases while the roundness of polyhedron with fewer surfaces grows more quickly. Although the initial polyhedron particles’ roundness varies greatly, after three abrasions, all of the particles’ roundness stabilize at around 0.91, which amply proves that the method described in this study can accurately simulate the abrasion process of polygonal particles.

(7) $$S_{3D}=\frac{A_{s}}{A_{p}}=\frac{4\pi \sqrt[{3}]{{\left(3V_{p}/4\pi \right)}^{2}}}{A_{p}}$$

## 3. Numerical Simulations

### 3.1. Sample Preparation

It is assumed that the particles in the four groups of specimens are nine forms in Figure 2 and Figure 5, with the same amount of particles in each form and a uniform distribution of particle size between 10 mm and 40 mm. As fragmentation of the particles is not taken into account in this study, the rigid block model in PFC6.0 [34] can be used to simulate the particles. The linear model in PFC6.0 is used for the inter-particle contact intrinsic model since the bonding force between particles is not taken into account. The initial porosity of different specimens varies because of the wide range of particle morphology. In order to make the results of each group of specimens comparable, a coefficient was used at the time of sample preparation, ensuring the greater relative density of the deposited granular material. Although there is contact sliding friction between the particles and the sidewalls [35], in the discrete element method (DEM), to avoid gradient changes in the internal stress of the sample and to produce specimens with a relatively uniform particle distribution, the sliding friction coefficient between the particles and the walls is set to zero. Studies have shown that this approach is feasible under low micropressure conditions [36]. The DEM parameters for simulations obtained from the previous calibration work [37] are shown in Table 1.

### 3.2. Conventional Triaxial Test 

**Figure 8:**
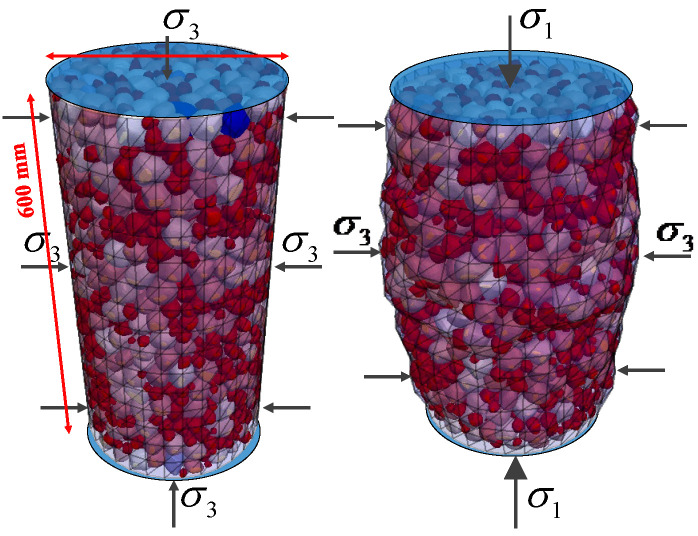
Conventional triaxial test of the DEM.

As shown in Figure 8, four groups of granular aggregates are generated in a frustum-shaped area of a cone with a base diameter of 300 mm and a height of 600 mm. The boundary is a shell-type element with a thickness of 2 mm and an elastic modulus of 7.8 MPa to simulate the rubber mold. The top and bottom loading plates are rigid wall elements. The interaction between the discrete particles and the lateral boundary can be realized by the discrete–continuous coupling method built into PFC6.0. A constant 100 kPa surrounding pressure $\sigma_{3}$ is applied to the sidewalls, and top and bottom loading plates are applied to all four groups of specimens after they reach a relative density of 0.8 in order to simulate the consolidation of the specimens. After the specimens reached stability, the confining pressure was kept constant, and the specimens were loaded by applying a relative velocity on the upper and lower loading plates, and the loading was stopped when the axial strain reached 15%. At the end of loading, an obvious uneven bulging deformation in the middle of the specimen was observed.

## 4. Macroscopic Mechanical Response

### 4.1. Deviatoric Stress and Strain 

In this study, a stress tensor is used to quantify the macroscopic mechanical response of granular materials in the shear process. The calculation method is as follows:

(8) $$\sigma_{ij}=\frac{1}{V}\sum_{c\in V}f_{i}^{c}l_{j}^{c}$$

(9) $$\begin{array}{l} p=\frac{1}{3}\sigma_{ii}\\ q=\sqrt{\frac{3}{2}\sigma_{ij}{\;\;}^{\prime }\sigma_{ij}{\;\;}^{\prime }} \end{array}$$

where ${\sigma^{\prime }}_{ij}$ is the deviator part of the stress tensor $\sigma_{ij}$, *V* is the volume of the specimen, $f_{i}^{c}$ is the component of the contact force vector ***f***, and $d_{j}^{c}$ is the component of the contact branch vector ***d***. Mean stress *p* and deviatoric stress *q* can be defined as:

The axial strain of the specimen is calculated as $\varepsilon_{a}=\ln\Delta H/H_{0}$, where ΔH is the loading displacement of the load plate and *H*_0_ is the initial height of the specimen. According to Ma’s [31] method, the volumetric strain $\varepsilon_{v}$ is determined by the relative placement of the loading plate and the lateral shell nodes during the loading process.

**Figure 9:**
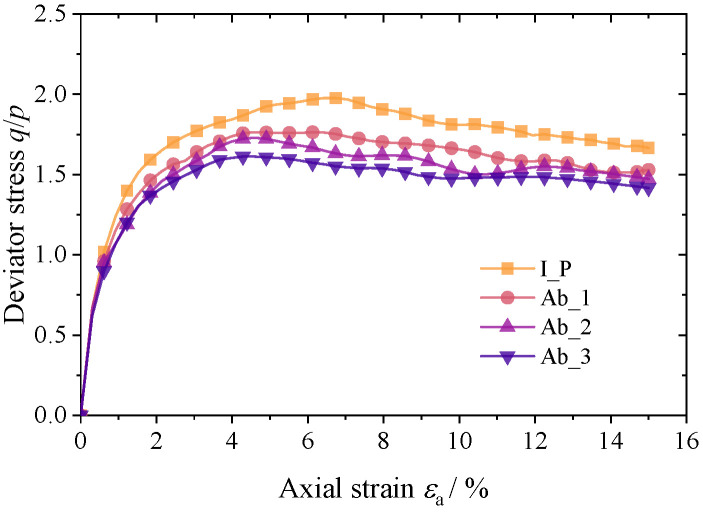
Axial stress–axial strain curves.

**Figure 10:**
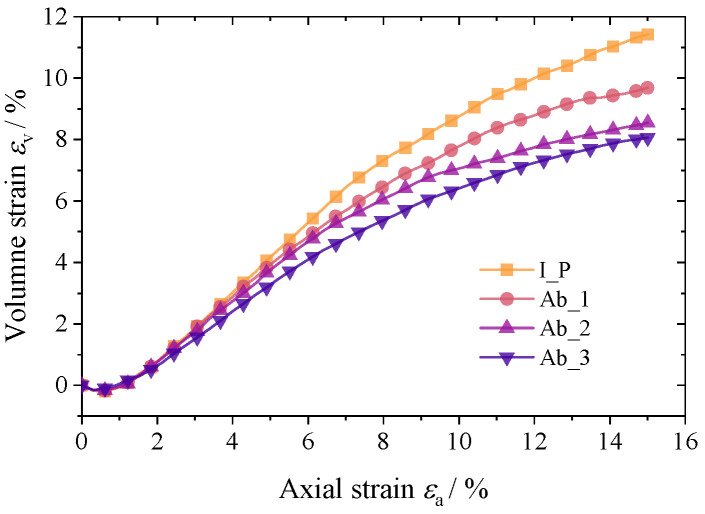
Volume strain–axial strain curves.

At a confining pressure of 100 kPa, Figure 9 and Figure 10 depict the macroscopic mechanical response of the four groups of materials. The simulation results demonstrate that the deviatoric stress–axial strain patterns are similar across all specimens and that after a brief linear increase at the start of loading, the growth rate quickly declines until the axial strain peaks at about 5%. The peak strength of the corroded granular specimens was marginally less than that of the initial polyhedron specimens, and it decreased as the degree of abrasion increased. At the same time, the dilatancy effect of particle specimens with a higher degree of angularity is more obvious, and increasing the abrasion degree of particles will inhibit the dilatancy of the material. The model test results show that this law is convincing for coarse sand materials composed of dense non-cohesive particles.

### 4.2. Strength and Deformation

**Figure 11:**
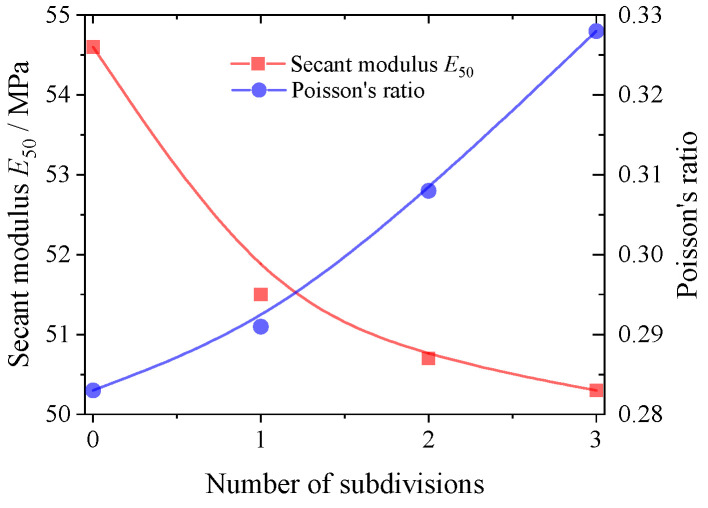
Variation in the secant modulus *E*_50_ and Poisson’s ratio *v* with the abrasion degree.

**Figure 12:**
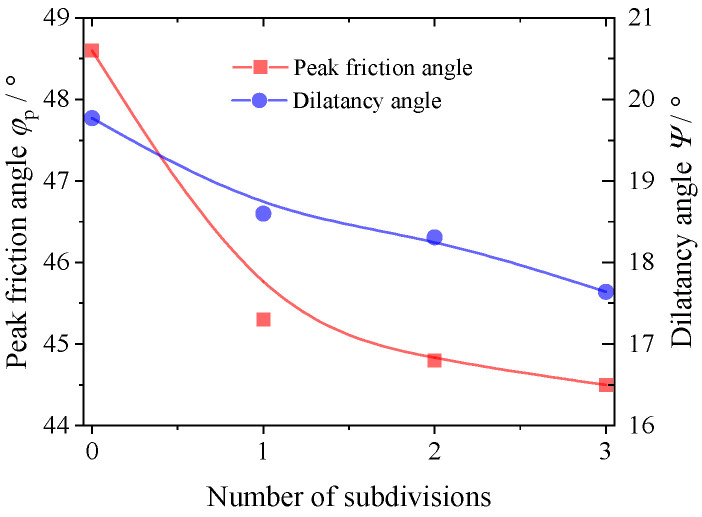
Variation in the peak friction angle *φ*_p_ and dilatancy angle *Ψ* with the abrasion degree.

Figure 11 and Figure 12 demonstrate the effect of the degree of particle abrasion on the secant modulus and strength of the granular material. The results of both figures show that the mechanical properties of the granular material are significantly influenced by its own particle shape. Increasing the degree of particle erosion weakens the modulus of the granular material and increases its Poisson’s ratio. The peak friction and shear expansion angles of the granular material decrease with increasing abrasion. This is due to the fact that the internal structure of the corroded granular material is more fragile and less able to resist external loads, a phenomenon that can be explained by the stress–dilatancy theory [38,39].

## 5. Meso Shear Properties

### 5.1. Coordination Number

**Figure 13:**
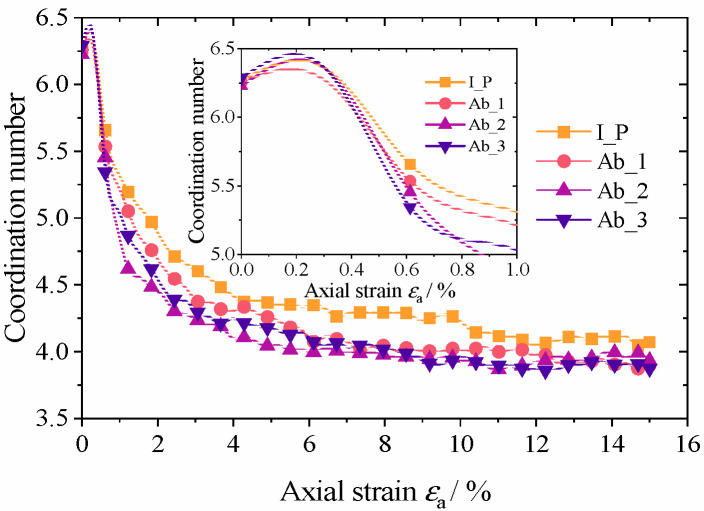
Mean coordination number during loading.

The coordination number is an important indicator in evaluating the microstructural properties and stability of granular materials; it is defined as the number of particles in contact with a given particle [40,41]. Usually, researchers are more concerned with the average coordination number of the particle system. The changes in the average coordination number of the four groups of specimens during the loading process are shown in Figure 13. The initial coordination number of the four sets of specimens was around 6.2, and the specimens showed a slight increase in the coordination number at the beginning of loading, indicating a slight shear contraction of the specimens. Subsequently, the average coordination number of the four groups of specimens decreased rapidly, but the rate of decrease in coordination number was proportional to the degree of particle abrasion. This indicates that the ability of the particle system to resist external loading decreases as the degree of abrasion increases. When the axial strain reaches about 11%, the coordination numbers of the four groups of tests remain stable. The critical coordination number, which is thought to be the minimum necessary required to maintain the specimen’s mechanical stability during the shear process, decreases slightly as the degree of abrasion increases but not noticeably. The critical coordination number for the four test groups remains constant between 3.8 and 4.1.

### 5.2. Internal Sliding and Rotation

**Figure 14:**
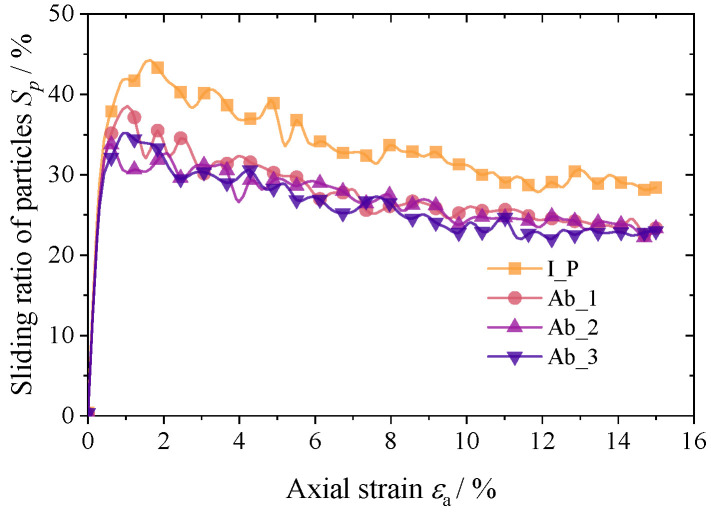
Mean sliding ratio during loading.

The macroscopic deformation of the specimen is manifested microscopically as the relative sliding and rotation between particles. Whether relative sliding between the particles occurs depends on the degree of friction play. The friction mobility index $I_{m}=\left|f_{t}\right|/\mu f_{n}$ can be used to judge whether sliding occurs between particles in contact with each other. When $I_{m}>0.9999$, relative sliding of the particles occurs, where fn and *f_t_* are the normal and tangential contact forces, respectively, and *μ* is the inter-particle friction coefficient. The sliding rate of the particle system is defined as $S_{p}=N_{sc}/N_{c}\times 100\%$, where *N_sc_* is the number of particles in which sliding occurs and *N_c_* is the total number of contacts. Figure 14 shows the variation in the sliding rate during loading for four groups of specimens. *S_p_* increases rapidly with increasing *ε*_a_ for the four groups of specimens and reaches a peak at $\varepsilon_{a}=1.5\%$. It then decreases slowly. Overall, *S_p_* increases more rapidly for the initial polyhedron group, and the average *S_p_* during loading is smaller as the abrasion increases.

**Figure 15:**
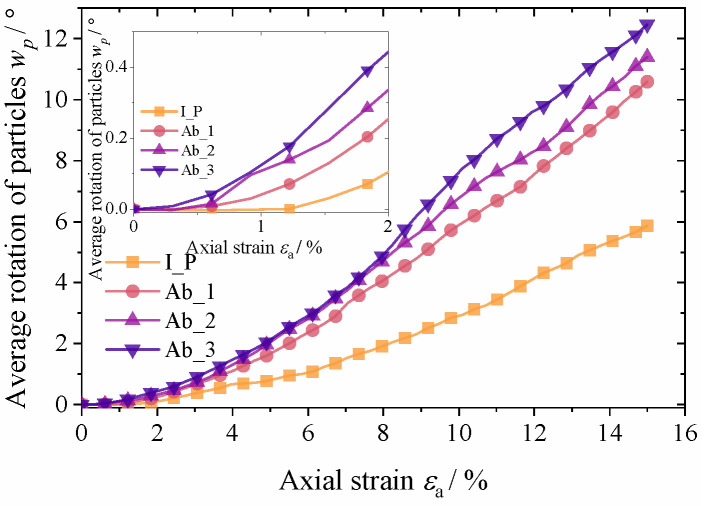
Average rotation angle of particles during loading.

Another microscopic cause of the deformation of the specimen is the rotation of the particles, and the average particle rotation *w_p_* is used here to describe the degree of particle rotation during the loading process. It is calculated as follows:

(10) $$w_{p}=\frac{1}{N_{r}}\sum_{a=1}^{N_{r}}\left|w_{a}\right|$$

where *w_a_* is the rotation angle of a single particle during loading and *N_r_* is the number of particles in the material. Figure 15 shows the average rotation angle of particles during the loading process in the numerical simulations described in Section 3. When ε_a_ was less than 1%, the initial polyhedron specimens produced almost no increment in rotation angle, while the group with the greatest abrasion produced significant rotation of the particles from the beginning of loading. Thereafter, the incremental rate of *w*_p_ in the four groups of specimens gradually accelerated during loading, and after *ε_a_* reached 8%, *w*_p_ increased linearly. The rate of increase in *w*_p_ increased significantly with the increase in abrasion degree, while the rate of increase in the rotation of the specimens in the initial polyhedron group was significantly lower than that of the other three groups. 

The results from the two experimental sets reveal the occurrence of mutual sliding and rotation between particles and indicate that the deformation of granular aggregates at low shear strain is primarily driven by relative sliding between particles. As shear strain increases, the percentage of material deformation attributed to particle rotation gradually increases. Specifically, in the low-roundness granular system, the percentage of deformation resulting from sliding is higher during shear deformation. This is attributed to the stronger interlocking effect among particles with lower roundness, which compels particles to slide in order to adjust the deformation of the specimen. Moreover, the particle system with lower roundness exhibits a larger average coordination number during loading, which enhances the particle resistance to rotation. These findings are consistent with the conclusions drawn by Estrada [42] and Azéma [43].

### 5.3. Fabric Anisotropy

(1) Anisotropy evaluation method

**Figure 16:**
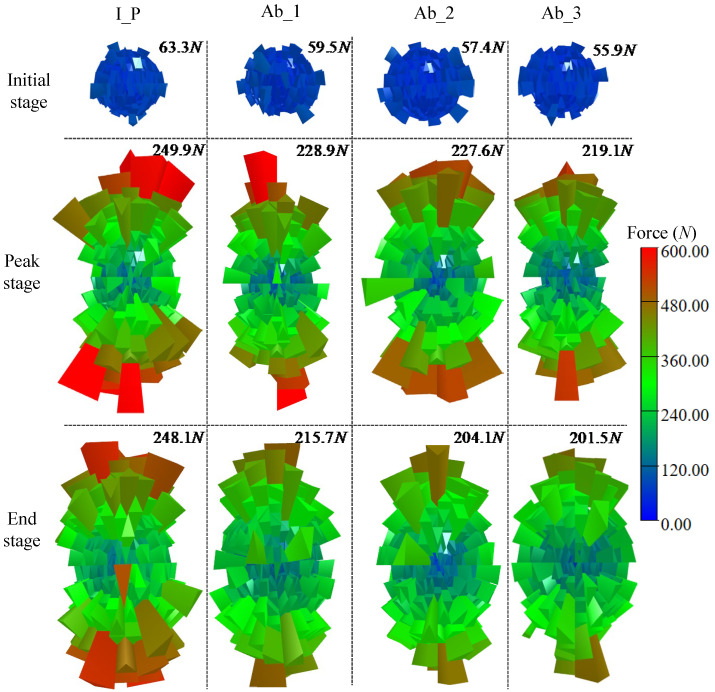
Column coordinate histogram of contact normal force.

Fabric, which refers to the macroscopic statistics of particle arrangement or contact, is the term used to describe the microstructure of granular materials [44,45,46]. The fundamental factor in determining the macro mechanical properties of granular materials is fabric anisotropy, a significant micromechanical and structural index of granular materials. The anisotropy of granular materials during loading has always been a research focus. Figure 16 shows the spatial distribution of the normal contact force in the granular system of four groups of samples before loading, at the peak state, and at the end of loading. The average contact force of each group of samples is marked on the upper right corner of the corresponding three-dimensional histogram. The normal contact force distribution of each group of samples is approximately isotropic under the same initial pressure in all directions. In the process of loading, the normal contact force of each group of specimens in the vertical direction increases fastest, indicating that shear causes the anisotropy of the normal contact force. Additionally, the rate of anisotropy is influenced by the degree of particle abrasion. The histogram becomes more slender as a result of the vertical normal contact force of particles with high angularity increasing more quickly during loading. The results demonstrates that the roundness of particles has a significant impact on the anisotropy brought on by shear, and the initial polyhedron sample’s average contact force is noticeably higher than that of the other samples. Based on the results in Figure 13, particles with higher angularity form tighter contact structures between particles. Furthermore, the results in Figure 15 indicate that more angular particles, because of their irregular shapes, can interlock better with each other. This interlocking effect increases the mechanical interlock force between particles, making it more difficult for the particles to slide relative to each other under external forces.

Quantitative indicators can be used to describe the anisotropy of granular materials. Anisotropy is typically divided into geometric and mechanical types. Geometric anisotropy refers to the fabric’s vector distribution of particle spacing, while mechanical anisotropy refers to the directivity of contact force under external load. Five fabric tensors are used to describe these indicators in order to analyze them quantitatively, including the fabric tensor of contact normal $\Phi_{ij}^{c}$:

(11) $$\Phi_{ij}^{c}=\frac{1}{N_{c}}\sum_{c\in V}n_{i}n_{j}$$

the fabric tensioners of normal and tangent branch vectors $\Psi_{ij}^{b_{n}}$ and $\Psi_{ij}^{b_{t}}$ due to irregular particle shape:

(12) $$\begin{array}{l} \Psi_{ij}^{b_{n}}=\frac{1}{N_{c}}\sum_{c\in V}\frac{b_{n}n_{i}n_{j}}{1+A_{kl}^{c}n_{k}n_{l}}\\ \Psi_{ij}^{b_{t}}=\frac{1}{N_{c}}\sum_{c\in V}\frac{b_{t}t_{i}n_{j}}{1+A_{kl}^{c}n_{k}n_{l}} \end{array}$$

and the fabric tensors of normal and shear contact forces $\Omega_{ij}^{f_{n}}$ and $\Omega_{ij}^{f_{t}}$ due to external forces:

(13) $$\begin{array}{l} \Omega_{ij}^{f_{n}}=\frac{1}{N_{c}}\sum_{c\in V}\frac{f_{n}n_{i}n_{j}}{1+A_{kl}^{c}n_{k}n_{l}}\\ \Omega_{ij}^{f_{t}}=\frac{1}{N_{c}}\sum_{c\in V}\frac{f_{t}t_{i}n_{j}}{1+A_{kl}^{c}n_{k}n_{l}} \end{array}$$

In Equations (11)–(13), *n_i_* and *t_i_* are the unit vectors in the contact normal and tangential directions, and *b_n_*, *b_t_*, *f_n_*, and *f_t_* are the projections of the branch vectors and contact force vectors in the contact normal and contact shear directions. The degree of contact anisotropy can be described by the deviatoric tensor $A_{ij}^{c}$ in Equation (19).

(14) $$A_{ij}^{c}=\frac{15}{2}\Phi_{ij}^{c^{\prime }}$$

The anisotropy evaluation method of normal and tangential branch vectors can be described by the deviatoric part of the tensor $A_{ij}^{b_{n}}$ and $A_{ij}^{b_{t}}$ in Formula (15):

(15) $$A_{ij}^{b_{n}}=\frac{15}{2}\frac{\Psi_{ij}^{{b^{\prime }}_{n}}}{{\bar{b}}_{0}}\;A_{ij}^{b_{t}}=\frac{15}{3}\frac{\Psi_{ij}^{{b^{\prime }}_{t}}}{{\bar{b}}_{0}}\;{\bar{b}}_{0}=\Psi_{ii}^{b_{n}}$$

Similarly, the anisotropy evaluation method of normal and tangential contact force anisotropy can be described by the deviatoric part of the tensor $A_{ij}^{f_{n}}$ and $A_{ij}^{f_{t}}$ in Equation (16):

(16) $$A_{ij}^{f_{n}}=\frac{15}{2}\frac{\Omega_{ij}^{{f^{\prime }}_{n}}}{{\bar{f}}_{0}}\;A_{ij}^{f_{t}}=\frac{15}{3}\frac{\Omega_{ij}^{{f^{\prime }}_{t}}}{{\bar{f}}_{0}}\;{\bar{f}}_{0}=\Omega_{ii}^{f_{n}}$$

where $\Psi_{ij}^{{b^{\prime }}_{n}}$, $\Psi_{ij}^{{b^{\prime }}_{t}}$, $\Omega_{ij}^{{f^{\prime }}_{n}}$, $\Omega_{ij}^{{f^{\prime }}_{t}}$ are the deviatoric parts of the fabric tensors in Equations (15) and (16), respectively.

The scalar value $A^{*}$ is introduced to describe the degree of fabric anisotropy. The calculation method is as follows:

(17) $$A^{*}=sign(A_{ij}^{*}\sigma_{ij}{\;\;}^{\prime })\sqrt{\frac{3}{2}A_{ij}^{*}A_{ij}^{*}}$$

where the superscript “*” indicates *c*, *b_n_*, *b_t_*, *f_n_*, and *f_t_* in Equations (11)–(17), *sign*() is a symbolic function, and ${\sigma^{\prime }}_{ij}$ is the deviatoric part of Equation (9). When the principal direction of $A_{ij}^{*}$ is consistent with the direction of the stress tensor, $sign(A_{ij}^{*}\sigma_{ij}{\;\;}^{\prime })$ is a positive number, and vice versa.

(2) Evolution of anisotropy

**Figure 17:**
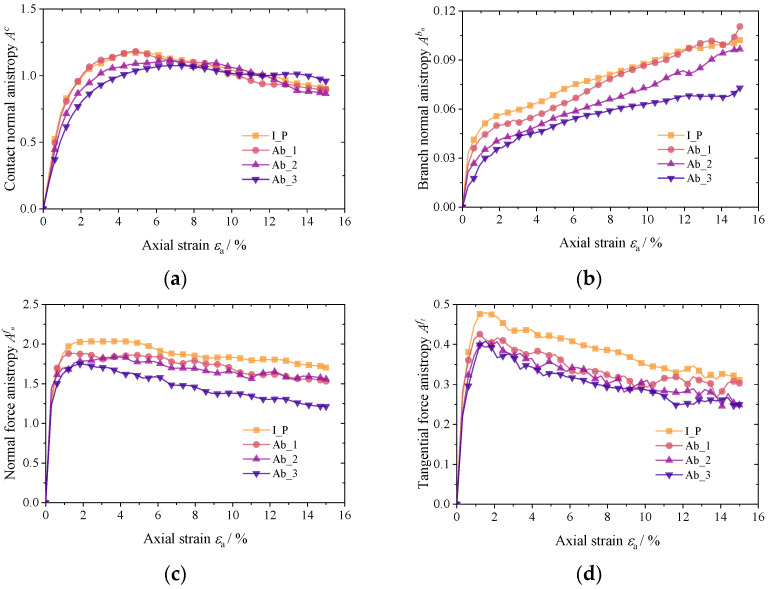
Evolution of anisotropy. (**a**) Contact normal vectors $A^{c}$; (**b**) normal branch vectors $A^{b_{n}}$; (**c**) contact normal force vectors $A^{f_{n}}$; and (**d**) contact shear force vectors $A^{f_{t}}$;.

The evolution patterns of $A^{c}$, $A^{b_{n}}$, $A^{f_{n}}$, and $A^{f_{t}}$ during loading are shown in Figure 17. Since there is not a distinct long and short axis for the particles in this study, the variation pattern in the $A^{b_{t}}$ term is not given here because of the minimal contribution of the branch vector in the contact shear direction (about 1%). The results show that the individual anisotropy exhibits softening characteristics as loading proceeds. In each group of results, the initial polyhedron particles have the highest degree of anisotropy, and as the abrasion level increases, the anisotropies all show varying degrees of decline. It is interesting to note that the anisotropy of $A^{c}$ and $A^{f_{n}}$ during loading closely resembles the granular system’s deviatoric stress. $A^{b_{n}}$ grows slowly in the loading process and does not show a significant peak, and the degree of anisotropy is far less than the other three groups. The shear force anisotropy $A^{f_{t}}$ first appears as a peak value in the loading process and rapidly decreases after the peak value.

(3) Stress–Force–Fabric relationship

According to some studies, the shear process of cohesionless granular materials and their fabric tensors have the following correspondence:

(18) $$\begin{array}{c} \sigma_{ij}=\frac{N_{c}{\bar{f}}_{0}{\bar{b}}_{0}}{3V}\left\{\delta_{ij}+\frac{2}{5}\left(A_{ij}^{c}+A_{ij}^{f_{n}}+\frac{3}{2}A_{ij}^{f_{t}}+A_{ij}^{b_{n}}+\frac{3}{2}A_{ij}^{b_{l}}\right)\right.\\ \left.+\frac{2}{35}\left[\left(A_{kl}^{f_{n}}-A_{kl}^{f_{t}}\right)A_{kl}^{c}\delta_{ij}+\left(4A_{il}^{f_{n}}+3A_{il}^{f_{t}}\right)A_{ij}^{c}\right]\right\} \end{array}$$

Equation (13) establishes the stress–force–fabric relationship, which is known as the relationship between the granular system’s fabric anisotropy and stress tensor. Considering that the anisotropy tensor product in square brackets is a second-order micro component, it can be omitted. Therefore, Equation (18) can be combined with Equation (9) to derive:

(19) $$a=\frac{q}{p}=\frac{\sqrt{\frac{3}{2}{\sigma^{\prime }}_{ij}{\sigma^{\prime }}_{ij}}}{\sigma_{ii}/3}≃\frac{2}{5}\left(A^{c}+A^{b_{n}}+\frac{3}{2}A^{{\;\;}_{b_{t}}}+A^{{\;\;}_{f_{n}}}+\frac{3}{2}A^{{\;\;}_{f_{t}}}\right)$$

Equation (19) demonstrates that the degree of anisotropy $A^{*}$ can be used to describe the deviator stress ratio. 

According to the measurement principle of the macro triaxial experiment, the confining pressure $\sigma_{3}$ is fixed at 100kPa and the normal stress $\sigma_{1}$ is defined as the total axial contact force of the particles acting on the loading plate divided by the area of the loading plate. The generalized average principal stress *p_g_* and deviatoric stress *q_g_* can be expressed as follows:

(20) $$\begin{array}{l} p_{g}=\frac{\sigma_{1}+2\sigma_{3}}{3}\\ q_{g}=\sigma_{1}-\sigma_{3} \end{array}$$

**Figure 18:**
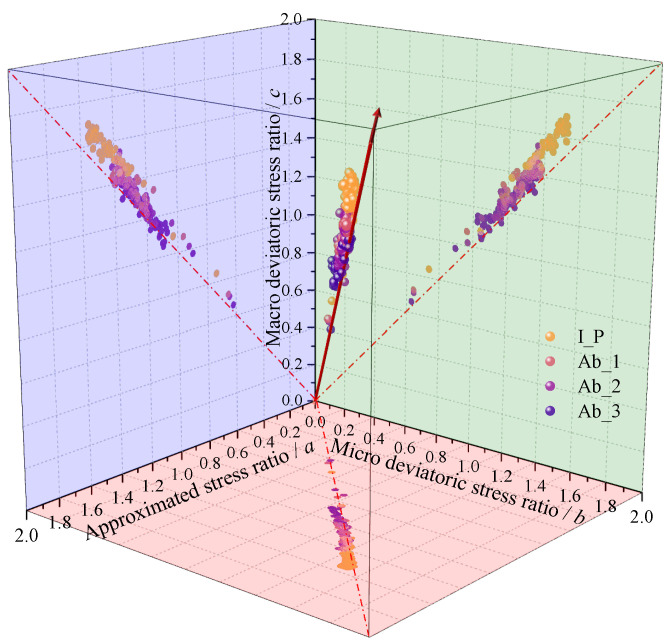
Validation in the stress–force–fabric relationship in terms of the deviatoric stress ratio for the 4 types of granular materials.

In Equation (20), the deviatoric stress ratio of the sample in the conventional triaxial test is $3(\sigma_{1}-\sigma_{3})/\left(\sigma_{1}+2\sigma_{3}\right)$. It should be noted that there are differences between the deviatoric stress ratios obtained using Equations (9) and (20). In contrast to Equation (20), which defines the shear stress ratio from a macro perspective, Equation (9) defines it from a micro contact perspective. The deviatoric stress ratio obtained by Equation (9) is denoted as *b*, while the one defined by Equation (20) is denoted as *c* for the purpose of distinction. In the three computational models based on Equations (19), (9), and (20), Figure 18 compares the variation in the deviatoric stress ratio throughout the loading process. The theoretical deviatoric stress ratio curve is represented in the figure as a red straight line. The deviatoric stress ratio scatter plot and the theoretical stress ratio straight line projection in the horizontal plane during the loading process of each group of tests are very similar, showing that the approximate deviatoric stress ratio calculated by Equation (19) and the theoretical deviatoric stress ratio of Equation (9) are in good agreement. The degree of anisotropy can be used to define the deviatoric stress ratio of the specimen. The large deviatoric stress ratio found in Equation (20) may be attributed to the specimen’s additional constraint by the rubber mold.

## 6. Results

In this study, a modeling method for random convex polyhedron particles is first developed, and subsequently, a particle abrasion method is proposed, which can better reflect the abrasion process of particles in their natural state. Based on the discrete element method, conventional triaxial tests are conducted on four granular aggregates with different abrasion degrees, and the macroscopic–microscopic connection between the granular materials is better captured. The main findings of this study are summarized as follows:

- A modeling method of randomly convex polyhedron particles was established. In addition, a method of generating different degrees of abrasion of the particles based on the corners and edges of the particles and the relative positions of the surfaces was established, which better reflects the abrasion process of the particles in their natural state. According to the results of the roundness index S3d, the particles have higher sphericity after abrasion. The particle abrasion function is derived from graphical and fundamental mechanical principles. However, it is necessary to couple it with the flow field to obtain a more detailed understanding of the particle abrasion mechanics.
- The macroscopic mechanical behavior of cohesionless granular materials is reproduced using traditional triaxial tests, including the nonlinear stress–strain relationship and dilatancy. With an increase in the degree of particle abrasion, the stiffness and macro shear strength of the material decrease, and the bulging deformation of the material is also restrained.
- The granular aggregate with a higher degree of abrasion has lower structural stability, a quicker decline in coordination number, and a lower average coordination number when achieving stability, according to microscopic analysis. At the initial loading stage, the granular material with a higher abrasion degree is more susceptible to relative rotation, while the granular aggregate with a lower abrasion degree is more susceptible to relative sliding. These micro mechanical phenomena can, to a certain extent, reveal the macro mechanical characteristics of granular materials.
- The major contribution to fabric anisotropy comes from normal contact force anisotropy, which is followed by contact normal anisotropy. The results of the S-F-F relationship for the four materials demonstrate that there is a good correspondence between the macro mechanical properties and the micro fabric of the materials and that this correspondence is independent of the particle abrasion level. The abrasion level has an inverse relationship with each fabric’s parameters. The additional constraint of the rubber mold will cause the internal friction angle of granular materials obtained from conventional triaxial tests to be overestimated even if the friction constraint between the particles and boundary is not taken into consideration.

## Figures and Tables

**Table 1:** Parameters for particles in the proposed model.

Parameter	Value
Particle density, *ρ* (kg/m^3^)	2600
Particle coefficient of friction, *μ_c_*	0.5
Boundary–particle friction *μ_b_*	0.0
Particle normal stiffness, *k*_n_ (N/m)	1 × 10^8^
Particle tangential stiffness, *k*_s_ (N/m)	5 × 10^7^
Damping coefficient, *d_p_*	0.3

## Data Availability

Data are contained within the article.
